# Structural Studies of a Lipid-Binding Peptide from Tunicate Hemocytes with Anti-Biofilm Activity

**DOI:** 10.1038/srep27128

**Published:** 2016-06-13

**Authors:** Osmar N. Silva, Eliane S. F. Alves, César de la Fuente-Núñez, Suzana M. Ribeiro, Santi M. Mandal, Diana Gaspar, Ana S. Veiga, Miguel A. R. B. Castanho, Cesar A. S. Andrade, Jessica M. Nascimento, Isabel C. M. Fensterseifer, William F. Porto, Jose R. Correa, Robert. E. W. Hancock, Suresh Korpole, Aline L. Oliveira, Luciano M. Liao, Octavio L. Franco

**Affiliations:** 1 Departamento Biologia, Instituto de Ciências Biológicas, Programa de pós-graduação em Genética e Biotecnologia, Universidade Federal de Juiz de Fora, Juiz de Fora–MG, 36036-900, Brazil; 2 SInova, Pós-graduação em Biotecnologia, Universidade Católica Dom Bosco, Campo Grande, MS, Brazil; 3 Laboratório de RMN, Instituto de Química, Universidade Federal de Goiás, C.P. 131, 74001-970 Goiânia, GO, Brazil; 4 Department of Microbiology and Immunology, University of British Columbia, Vancouver, British Columbia, Canada; 5 Central Research Facility, Department of Chemistry and Department of Biotechnology, Indian Institute of Technology Kharagpur, India; 6 Instituto de Medicina Molecular, Faculdade de Medicina, Universidade de Lisboa, Av. Prof. Egas Moniz, 1649-028 Lisbon, Portugal; 7 Programa de Pós-Graduação em Inovação Terapêutica, Universidade Federal de Pernambuco, 50670-901 Recife, PE, Brazil; 8 Department of Cellular Biology, Institute of Biological Sciences, University of Brasilia, Brasilia, 70910900, Brazil; 9 Instituto de Química, Universidade de Brasília, C.P. 04478, 70910-000, Brasília, DF, Brazil; 10 Programa de Pós-Graduação em Ciências Genômicas e Biotecnologia, Centro de Análises Proteômicas e Bioquímicas, Universidade Católica de Brasília, Brazil

## Abstract

Clavanins is a class of peptides (23aa) histidine-rich, free of post-translational modifications. Clavanins have been studied largely for their ability to disrupt bacterial membranes. In the present study, the interaction of clavanin A with membranes was assessed by dynamic light scattering, zeta potential and permeabilization assays. We observed through those assays that clavanin A lysis bacterial cells at concentrations corresponding to its MIC. Further, the structure and function of clavanin A was investigated. To better understand how clavanin interacted with bacteria, its NMR structure was elucidated. The solution state NMR structure of clavanin A in the presence of TFE-d_3_ indicated an α-helical conformation. Secondary structures, based on circular dichroism measurements in anionic sodium dodecyl sulfate (SDS) and TFE (2,2,2-trifluorethanol), *in silico* lipid-peptide docking and molecular simulations with lipids DPPC and DOPC revealed that clavanin A can adopt a variety of folds, possibly influencing its different functions. Microcalorimetry assays revealed that clavanin A was capable of discriminating between different lipids. Finally, clavanin A was found to eradicate bacterial biofilms representing a previously unrecognized function.

The larvae of protochordates, such as the solitary tunicate *Styela clava*, display an assemblage of phenotypes that reveal their relationship with vertebrates^1^, including the presence of a post-anal tail, pharyngeal gill slits, a dorsal tubular nerve cord and a notochord. Furthermore, the body cavity and tissues contain phagocytic cells named hemocytes, which resemble vertebrate granulocytes and macrophages^2^. In *S. clava*, hemocytes synthesize two families of antimicrobial peptides, namely styelins and clavanins^3^. While styelins are larger peptides (~3.6 kDa) that contain hydroxylysines and other modified residues^4^, clavanins are the smallest known histidine-rich peptides, composed of only 23-amino-acid residues in length and having no post-translational modifications^5^.

In recent years, clavanins have been studied with particular attention to their ability to form pores in and disrupt model membranes^6^,^7^,^8^. Indeed, some studies suggest that clavanin A (VFQFLGKIIHHVGNFVHGFSHVF-NH_2_) is efficiently inserted into different phospholipid monolayers in a pH-dependent manner, mainly as a result of hydrophobic interactions. For example, van Kan *et al*.^7^ observed that, at neutral pH, wild-type clavanin efficiently released fluorophores from unilamellar vesicles, thus indicating a nonspecific permeabilization mechanism. However, clavanin A was unable to permeabilize model bilayers at low pH, suggesting that its activity could be modulated under different conditions, as observed for certain broad-spectrum antimicrobial peptides from plants^9^. Nevertheless, it has been suggested that all cationic amphipathic peptides disrupt membranes at sufficiently high concentrations^10^,^11^. Therefore, it was important to determine how peptide fold patterns varied in different environments and how this influenced their functions. Despite previous efforts, very little is known about the structure of clavanin A and its functions other than its antimicrobial property.

The data presented in this study represent the first description of the structure of clavanin A, its interaction with lipids, and present a novel activity for this peptide: its ability to eradicate biofilms in a broad-spectrum manner. These studies included interaction assays of clavanin A with liposomes and lipids, elucidation of the NMR structure of clavanin A, *in silico* docking and molecular dynamics, and using a flow cell chamber method to characterize the anti-biofilm activity of the peptide.

## Results

### Evaluation of clavanin A and lipid membrane interactions

**Figure 1:**
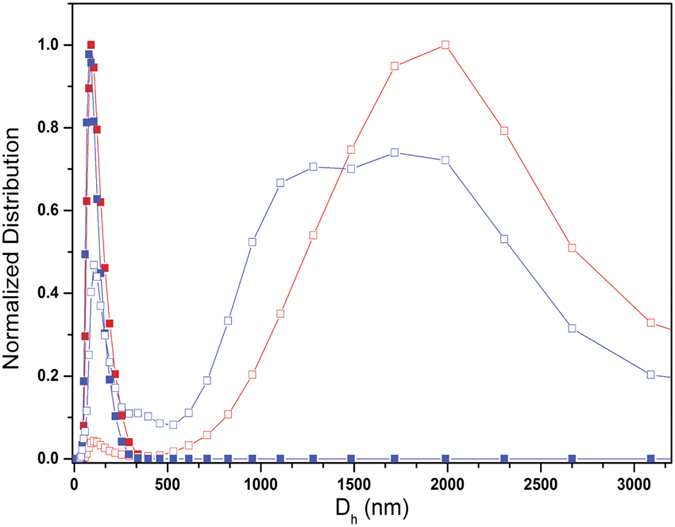
Determination of the diameter of unilamellar lipid vesicles (ULV). The hydrodynamic diameter (Dh) of lipid vesicles in the absence (solid squares) and presence of clavanin A (empty squares) as assessed by dynamic light scattering. In the absence of peptide, both POPC (red) and POPC:POPG (blue) vesicles had a narrow distribution of size centred at 110 nm. When the peptide was present, there was evidence of higher association with the anionic POPC:POPG vesicles (less residual 110 nm liposomes) as well as a large amount of aggregation and multimodal distributions, likely due to fusion, that were detected.

Membrane interactions are considered to be important components of the action of amphipathic antimicrobial peptides on bacterial cells^12^. Mammalian membranes are predominantly composed of neutral lipids, while bacterial membranes contain a net negative charge due to a substantial proportion of negatively charged lipids. The interaction of clavanin A at 10 μM with neutral (POPC) and negatively charged (POPC:POPG, 8:2) LUVs was studied using assessment of ζ-potential (Table 1) and dynamic light scattering (DLS). ζ-potential measurements indicated that there was a small neutralization of surface negative charge on liposomes (Table 1). In the absence of peptide, dynamic light scattering measurements (Fig. 1) showed that the LUVs had a mean diameter of 100 nm. Addition of clavanin A, however, at a peptide:lipid ratio of 1:100, led to larger hydrodynamic ratios (mean diameter of 1900 nm and 1550 nm for POPC and POPC:POPG, respectively) (Table 2). The peptide clearly bound better to the anionic liposomes as revealed by the smaller residual 110 nm peak. Overall these results demonstrated the direct interaction of clavanin A with liposomes leading to liposome fusion.

At higher concentrations, experiments assessing membrane leakage induced by clavanin A, based on detection of increased fluorescence due to dye release, were performed according to the method described by Chen and Chen^13^.

**Figure 2:**
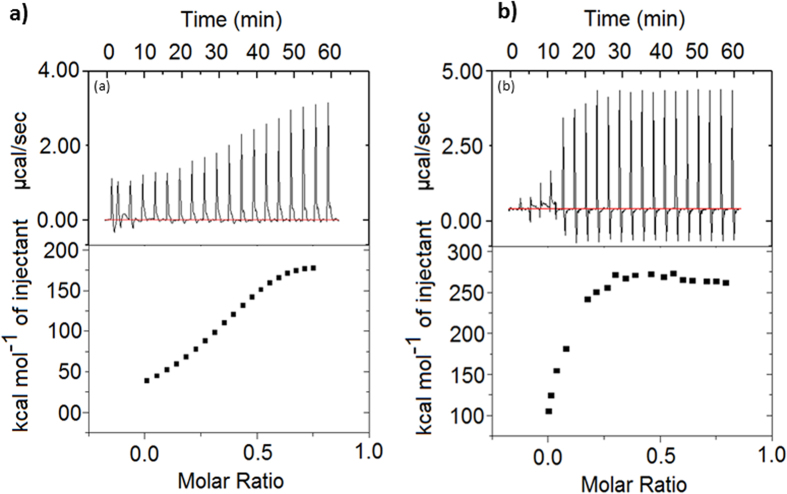
ITC binding experiments of clavanin A with lipid membrane components such as DOPC and DPPC. Displayed are raw heat-associated thermograms (upper panel) and isotherms (lower panel) for each individual set of interactions were displayed. The panels represent the interactions of clavanin A with DOPC (**a**) and DPPC (**b**). These data were corrected by subtraction of appropriate blank experiments and then fit using nonlinear regression. The conclusions derived from these analyses are presented in Table 2.

To further study the affinity of clavanin A towards phospolipids, microcalorimetry analyses were performed (Fig. 2), showing concentration-dependent binding and endothermic processes. Clavanin A had a greater effect at lower concentrations to vesicle of POPC:POPG mixture at 8:2 molar compared to only POPC. During interaction, it was observed that the ΔG value was negative, indicating that complex formation was energetically favourable and the process was entropy-driven for neutral lipid, POPC and negatively charged vesicles (POPC:POPG), which suggested that the hydrated clavanin A released bound water molecules during its interaction with lipid molecules. The positive values of ΔH and ΔS indicated that the interaction was driven by hydrophobic forces between the peptide and phospholipids. Higher ΔS value for vesicle (POPC:POPG) was observed compared to POPC and the peptide showed better binding affinity to negatively charged vesicles than POPC (Table 3).

### Clavanin A structure

**Figure 3:**
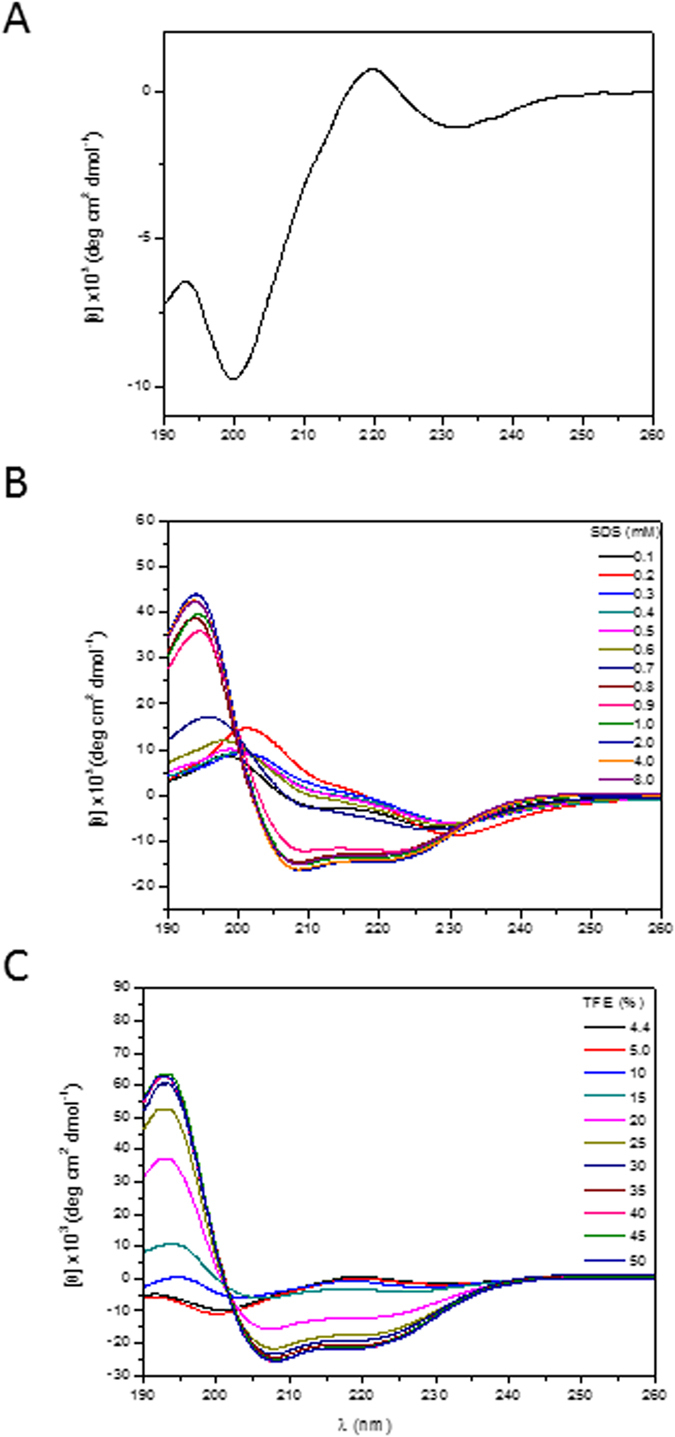
Circular dichroism spectra of 41 μM clavanin A, pH 5.0, at 25 °C. Spectra are in aqueous solution (**A**), and in different concentrations of SDS (**B**) and TFE (**C**).

Far-UV CD spectra of clavanin A (Fig. 3A) showed that the peptide had a random conformation in water, which was implied by the presence of a negative band at 200 nm. The addition of 1 mM SDS micelles (an anionic lipid mimic) (Fig. 3B) or 20% the solvent TFE (Fig. 3C) induced a conformational transition in the peptide to an alpha-helical structure, as shown by an intense maximum at 194 nm and two minima at 208 and 222 nm. Variation of the pH in the environment did not significantly change the conformation (data not shown). The thermal denaturation curves analyzed from CD measurements at 222 nm and pH 5 strongly indicated that clavanin A was a thermally unstable peptide, since spectra consistent with peptide denaturation could be observed (data not shown).

**Figure 4:**
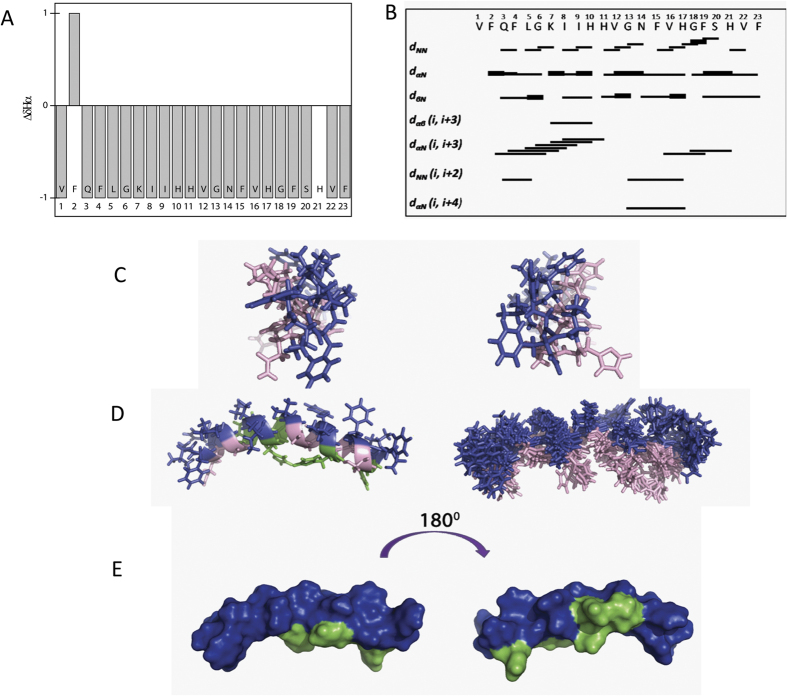
Clavanin A NMR analyses. (**A**) Chemical Shift Index. Shown are differences between the observed chemical shifts of protons (Hα) and unstructured value patterns (42). (**B**) Summary of NOEs used for structure calculation. The thickness of the bars indicates the relative intensities of the NOEs (strong, medium and weak). (**C**) Front view of N-terminal (left) and C-terminal (right) 3D structure. (**D**) Lowest energy structure (left) and backbone superimposition of 10 lowest energy structures (right). Color indicates: pink - polar, green- cationic and hydrophobic, blue - residues. (**E**) Surface structure. Cationic residues are indicated in green. The structure on the right side shows the molecule rotated 180° around the vertical axis.

The structure of clavanin A was investigated by solution-phase NMR spectroscopy in 35% TFE, since CD studies indicated an elevated α-helical content under these conditions and similar spectra were observed in the anionic lipid mimic SDS. There are reports in the literature that the 2,2,2-trifluoroethanol is a solvent that promotes the formation of hydrogen bonds in regions of the peptide α-helical where there is tendency, inducing structure only where there is already a tendency of forming the same^14^,^15^. Therefore, due to the limited resolution obtained in experiments using SDS-d_25_ TFE-d_3_was used as environment structuring inductor. The complete ^1^H resonance assignments of the backbone and side-chains of clavanin A were obtained using standard sequential assignment procedures. The elements of secondary structure of the peptide were predicted by the chemical shift index (CSI)^16^, which showed the deviation of the Hα chemical shifts from the corresponding amino acid embedded in a random coil structure. The presence of at least four consecutive “−1” values indicated the presence of a helical conformation. Figure 4A indicates the presence of anα-helical conformation between residues Gln3-Phe23. The patterns of the NOEs (Fig. 4B) also provide a qualitative indication of their secondary structure. The data showed a pattern characteristic of anα-helix between residues Phe2-His11 and Val16-His21. Only one restriction of Hα(i)-NH(i + 4), between residues Gly13-His17, was observed and no Hα(i)-NH(i + 3) spin coupling (NOEs) was found between residues His11-Val16. The NMR structure of clavanin A was calculated using an average of 12.3 restraints for each amino acid residue, including 42 dihedral angles obtained from ^13^C and ^15^N chemical shifts and was refined to a final RMSD of 1.05 ± 0.34 Å (backbone). Statistical analyses are described in Table 4. The NMR structure revealed an amphipathic α-helical conformation spanning residues Phe2 to Val22 (Fig. 4C–E). The 3D structure of the peptide in TFE-d_3_ conformed with a well-defined 〈-helical segment, which was mildly distorted by a slight curvature in the central region between residues Gly13 and His17. These results were consistent with the CSI and NOE data.

The Ramachandran plot indicated the good quality of the NMR models, highlighted by the fact that 100% of φ and ψ dihedral angles were in the favored allowed regions of α-helix^17^. These results were validated by QUEEN analyses, which indicated both the degree of agreement of the model structures with the experimental data, as well as the quality of their geometrical properties. The refined final structures in 35% TFE-d_3_ showed no errors and were well supported by the dataset (data not shown).

### DOPC and DPPC lipids stabilized the clavanin A structure in a water environment

**Figure 5:**
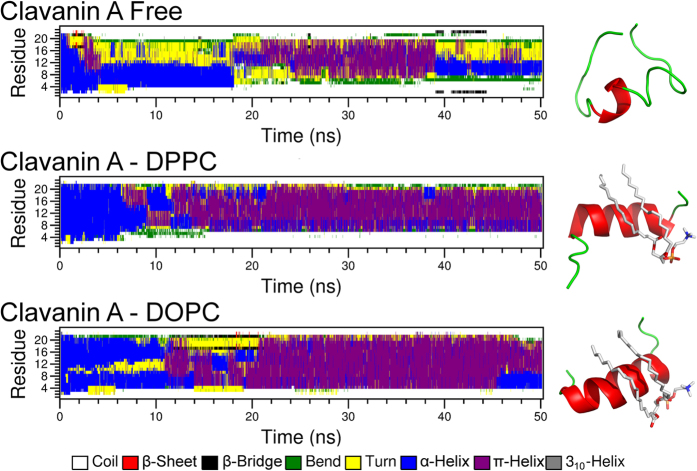
Standardized secondary structure assignment (DSSP analysis) based on molecular dynamics simulations after 50 ns of simulation starting with the NMR structure. The free clavanin A underwent an unfolding process, while in complex with lipids the π-helix is stabilized.

**Figure 6:**
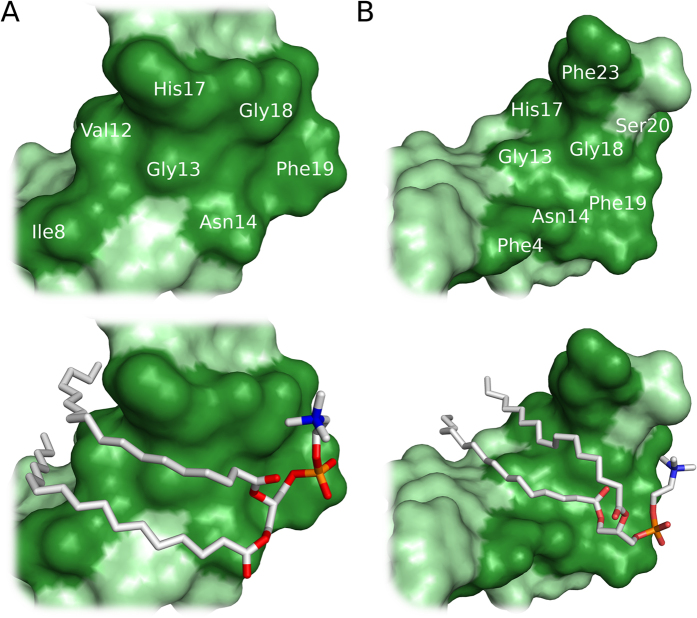
Predicted conformation of clavanin A interacting with DOPC (**A**) and DPPC (**B**). This shows the residues of clavanin A that interact with the lipids. The dark green regions indicate the residues with close contacts to lipids (less than 3.7 Å).

Molecular dynamics simulations were performed to assess the interactions between clavanin A and lipids DOPC and DPPC. It was observed that when the α-helical clavanin A was added to water, the peptide unfolded, with a clear transition from an α-helix to a coiled structure (Fig. 5). Nevertheless, when added to DOPC and DPPC, clavanin A was stabilized in a π-helix structure (4.1 residues per turn) with flexible termini (Fig. 5). This difference between the simulations could also be observed through the evolution of RMSD along the simulations, where the free peptide assumed a root mean square deviation (RMSD) of around 8 Å, while the peptide in complex with DOPC and DPPC had tighter RMSD values of around 5 and 4 Å, respectively (data not shown). Despite the observation that clavanin A underwent an unfolding process in water, the region comprised of residues ^9^IHHV^12^ remained in an α-helical conformation (Fig. 6). In interacting with lipids, the helical structured regions (considering both α- and π-helix) were larger, comprising the residues ^6^GKIIHHVGNFVHGFS^20^ and ^4^FLGKIIHHVGNFVHGF^18^, for the DPPC and DOPC complexes, respectively (Fig. 6). In addition, both lipids bound preferably to the C-terminal region of the peptide. The binding to DOPC was stabilized by residues Ile8, Val12, Gly13, Asn14, His17, Gly18 and Phe19 (Fig. 6), while for DPPC, the residues that stabilized the interactions were Phe4, Gly13, Asn14, His17, Gly18, Phe19, Ser20 and Phe23 (Fig. 6).

### *In vitro* activity against pre-formed biofilms

**Figure 7:**
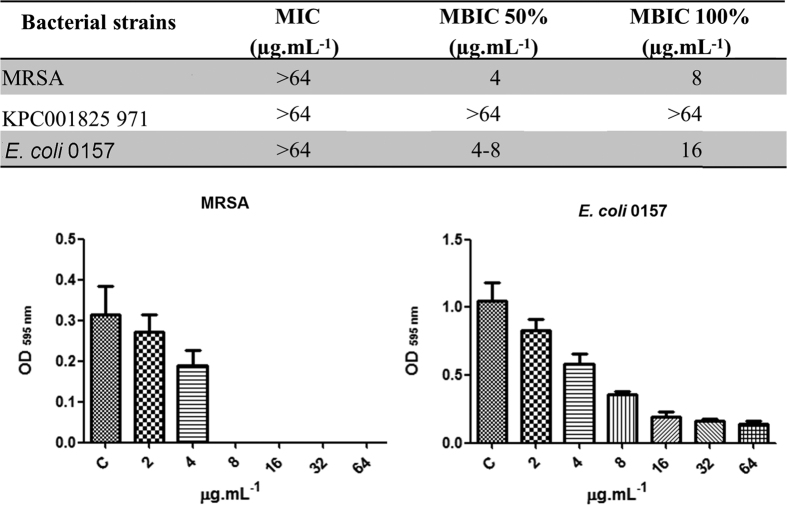
Evaluation of the antimicrobial and antibiofilm activity of Clavanina A against MRSA, *K. pneumoniae* 1825971 (KpC isolate) and *E. coli* (O157). MIC, minimal inhibitory concentration; MBIC, minimal biofilm inhibitory concentration; (**C**) control growth (bacteria withouth any treatment). Clavanin exhibit a dose-dependent anti-biofilm activity against E.coli and MRSA.

**Figure 8:**
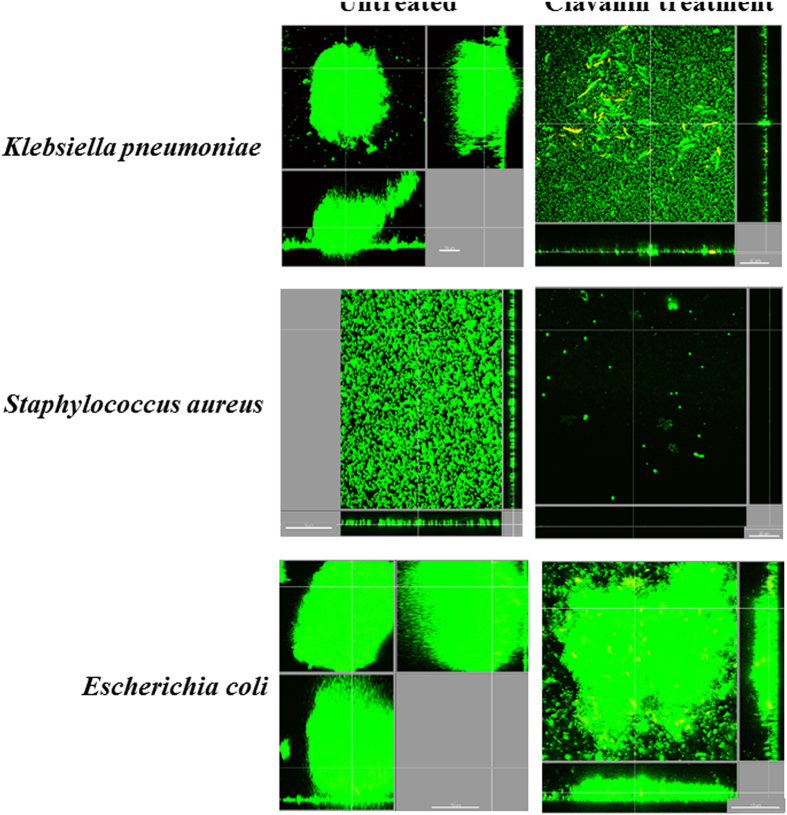
Anti-biofilm evaluation of clavanin (**A**). Flow cell assays were performed by using peptide concentration at a standard concentration of 16 μg.ml^−1^. Left and right panels correspond to untreated and peptide-treated *Escherichia coli*, *Klebsiella pneumoniae* and *Staphylococcus aureus*. Clavanin A (16 μg.mL^−1^) was able to disturb pre-formed (2 days old) biofilms of MRSA and KPC-*K. pneumoniae* 1825971 in flow cell experiments, eliminating biofilm formation completely for the MRSA strain and strongly reducing the thickness of *K. pneumoniae*.

**Figure 9:**
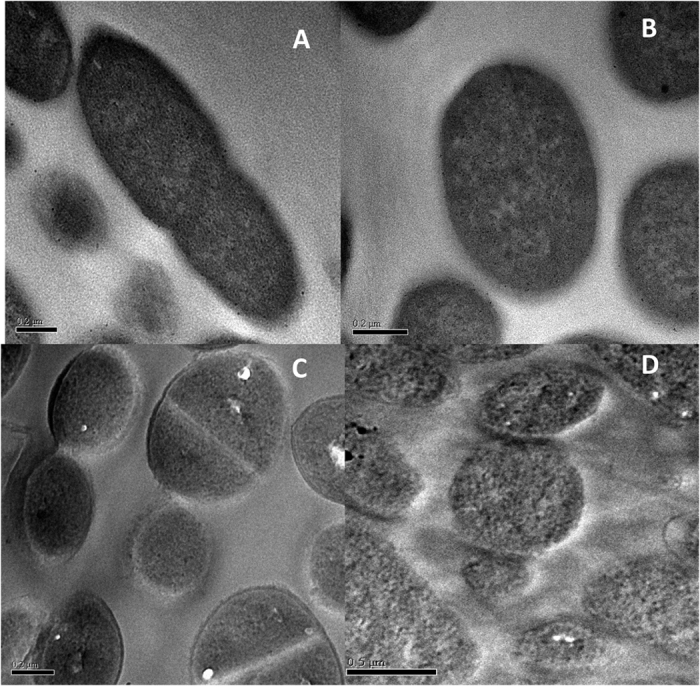
Transmission electronic microscopy of bacterial cells challenged by clavanin. (**A**) *Escherichia coli* was evaluated in the absence (**A**) and in the presence (**B**) of 32 μg.ml^−1^ clavanin A. *Staphylococcus aureus* was evaluated in the absence (**C**) and in the presence (**D**) of 64 μg.ml^−1^ clavanin A. Both bacteria tended to filament upon clavanin A exposure. Black bars correspond to 0.2 μm.

Clavanin A was chemically synthesized and further evaluated with regards to its primary structure by MALDI-ToF analyses, revealing a major peak with a molecular mass of 2,665.43 Da (data not shown). In our MIC assays using BM2 glucose minimal medium, clavanin A exhibited modest direct antimicrobial activity with MIC values of >64 μg.ml^−1^ against *E. coli* 0157, *K. pneumoniae* 1825971 (KPC-KP, isolate) and the Gram-positive organism methicillin-resistant *S. aureus* (Fig. 7). We also evaluated the anti-biofilm activity of Clavanin A. We observed through microplate assays that Clavanin A inhibited the biofilm formation of MRSA (8 μg.mL^−1,^ MBIC 100% and *E. coli* O157 (16 μg.mL^−1^ MBIC 100%) at concentrations lower than its MIC (>64 μg.mL^−1^). Using flow cell method, we observed that clavanin A, at 16 μg.mL^−1^, successfully perturbed pre-formed (2 days old) biofilms formedof *K. pneumoniae* 1825971 (KPC-KP isolate) and MRSA (Fig. 8). On the other hand, treatment with the peptide only reduced the overall biofilm thickness of *E. coli* 0157 pre-formed biofilms (Fig. 7). While performing these experiments, we observed that clavanin A at times induced filamentation in *K. pneumoniae* 1825971 (KPC-KP isolate) (Fig. 9). Experiments using transmission electron microscopy also revealed filamentation in cells of *S. aureus* and *E. coli* with one half of the peptide MIC. Fig. 8). Although dead cells were observed, around 50% of all *E. coli* cells appeared to be undergoing cell division (Fig. 9B).

Experiments using transmission electron microscopy showed that untreated cells of E. coli (Fig. 9A) and S. aureus (Fig. 9C) showed a normal cellular shape with an undamaged structure and slightly waved outer membrane. After incubation with a sub-MIC clavanin A concentration, it was observed the formation of filamentation in cells of E. coli (Fig. 9B) and S. aureus (Fig. 9D).

## Discussion

Clavanins are part of the defense system of sea tunicate *S. clava* against pathogens, and their antimicrobial and membrane disruptive activities have been extensively studied^8^,^19^. However, comprehensive structural studies on these peptides have not been carried out. Here, we focused on peptide clavanin A and performed an array of different assays aimed at providing insights about the structure and interactions of this peptide. Since lipids are the classical target of clavanin A^7^, ITC analyses were performed that clearly showed the ability of clavanin to discriminate between different lipids (Fig. 2). Moreover, in order to shed some light over the possible mechanism of action of clavanin A, dynamic light scattering and ζ-potential were performed to evaluate the interaction of the peptide with membranes. Addition of clavanin A led to the formation of large structures indicating its interaction with lipid vesicles to cause fusion. This effect was even more pronounced with POPC compared to POPC:POPG, despite apparently lower interaction with the uncharged POPC liposomes (Figs 1 and 2). Clavanin A is a peptide rich in lipophilic amino acid residues, favoring its interaction with lipid bilayers by means of hydrophobic interactions. This was also suggested by micro calorimetric titration. Diverse AMPs are capable of triggering aggregation of lipid vesicles since they interact with neutral lipids via hydrophobic interactions and neutralize liposome charges^19^,^20^,^21^. For example, peptides analogous to trichogin interact with neutral phospholipids and cause vesicle aggregation due to hydrophobic interactions^21^. Moreover, clavanin A was also shown here to promote membrane leakage consistent with the results of Van Kan *et al*.^6^. Clavanin A showed the ability to permeabilize the membranes of vesicles even at modest concentrations. The release of encapsulated solutes induced by AMPs occurs either through transient channels formed due to membrane perforation^22^. However, the mechanism of action of clavanin is likely to be multifunctional^8^, consistent with our results presented in this study and the observation that sub-MIC levels of the peptide that do not cause membrane disruption, induce cell filamentation (consistent with cell division inhibition) in some cases (Fig. 8). This effect on bacterial morphology may be due to interference of clavanin A with the cell division machinery, which will be the focus of future investigations. Interestingly, an analogous observation was made by Rosenberger *et al*.^23^ as they reported that *in vitro* incubation of *Salmonella enterica* serovar Typhimurium with sub-lethal levels of mouse cathelicidin-related antimicrobial peptide (CRAMP) triggered cell filamentation with arrested septum formation; while Friedrich *et al*.^24^ showed similar observations with other peptides.

In the present study, we have shown the interaction of clavanin A with different lipids. But how exactly does clavanin A bind to lipids and what are the forces involved in these interactions? To clarify this issue, CD and NMR analyses were performed solving the structure of clavanin A in solution. In the absence of hydrophobic conditions, random conformations were observed for the peptide which changed in the amphipathic environment where adopted a helical structure. These data shed some light over previous CD experiments, where an increase in the molar ellipticity at 222 nm of clavanin A occur in the presence of small unilamellar vesicles of DOPC (Kan *et al*.^7^), and also α-helical formation of clavanin A in membranes of DOPG:DOPC (1:9) (Kan *et al*.^6^). Other AMPs have been shown to undergo similar structural transitions. For example, the polar fish multifunctional peptide Pa-MAP1^25^,^26^, a conformational transition was detected in which a random fold in water and a full α-helical core was observed in SDS and TFE environments.

Structural calculations showed that the lowest energy structures obtained for clavanin A had a break helix in Gly13 (data not shown). Thus, to obtain better resolved structures ^13^C HSQC and ^15^N HMBC experiments were performed, through which it was possible to determine the φ and ψ torsion angle of the main chain, using TALOS+. After dihedral angles addition, the peptide presented a curved structure centered in this residue (Gly13). Magainin2, is other antibiotic peptide that also contains 23 amino acid residues and curved helical structure centred on residues Phe12 and Gly13^27^.

Pukala and collaborators^28^ showed the importance of structural flexibility in the activity of caerin 1.1, a potent broad-spectrum antibiotic peptide. In the membrane mimetic environment, this peptide adopts two α-helices structures separated by a flexible hinge region delimited by Pro15 and Pro19. The importance of the two Pro residues was assessed by replacement for Ala or Gly. The resulting structures indicate that the central angle of fold and its activity decreased significantly after replacing the Pro residue by Gly and more drastic reduction was observed when Pro was replaced by Ala. Thus, highlighting the need for structural curvature in its biological function. These relationships indicate that the flexible hinge allows an optimal orientation in any N- and C-terminal region of the peptide when it interacts with the membranes of the bacterial cells^29^,^30^.

Van Kan *et al*. observed that substituting Gly13 and Gly18 or only Gly18 with Ala generated more potent peptides against *Micrococcus flavus*. On the other hand, substituting only Gly13 led to slightly less potent peptides, while substitutions in Gly6 almost completely abolished peptide activity^6^. Therefore, Gly6 seems to be important for peptide flexibility, Gly18 for lipid interaction and Gly13 for both. In summary, it seems that are a variety of conditions including the type of lipid, pH^6^, and concentration that could directly affect the structural and functional properties of clavanin A.

In this context, by NMR, our work demonstrate that clavanin A adopts a curved α-helical conformation due to flexibility provided by residues of Gly, specially due to Gly-13, endorsing the importance of structural curvature demonstrated by the above authors.

Clavanins show some structural and functional homology to magainin 1, a well-characterized antimicrobial peptide from the skin of *Xenopuslaevis* that also shows such structural transitions^31^. Despite the fact that magainins are lysine-rich and clavanins histidine-rich peptides, 15 amino acid residues of clavanin A (located between Leu5 and Phe19) show 12 similarities (6 identical residues and 6 conservative substitutions) with residues present between His2 and Phe16 in magainin 1^5^. According to the results of molecular dynamics simulations this region of clavanin A with high similarity to magainin 1 was the major contributor to interaction of this peptide with lipids DOPC and DPPC (Fig. 6).

In simulations, residues Gly13, Asn14, His17, Gly18 and Phe19 had close contacts with lipids. These data clarify the results obtained by van Kan *et al*.^6^ in experiments replacing Gly for Ala variants, as described above. Indeed, Franco^9^ proposed that multifunctional and promiscuous peptides are not only influenced by their structures but also to micro-environmental conditions that could drive peptide folding and interaction with bacteria, and therefore its activity. This concept is consistent with the data reported here that suggest that the activity of clavanin A may depend on which lipid(s) with which it interacts, consistent with previous studies focusing on other linear AMPs such as magainin^32^ and LL-37^33^ that indicate that their activity can be governed by micro-environmental properties.

Finally, flow cell experiments revealed that treatment with 16 μg.mL^−1^ of clavanin A (i.e., one quarter it’s MIC) effectively eradicated pre-established biofilms formed by KPC-*K. pneumoniae* 1825971 and MRSA, and reduced the thickness of *E. coli* 0157 biofilms. These results identify a novel, previously unrecognized role for clavanin A as an anti-biofilm peptide. The amino acid sequence of clavanin A provides a good template for anti-biofilm activity that will serve as the basis for subsequent structure-activity relationship studies aimed at producing synthetic variants with improved activity against biofilms.

In conclusion, we have provided novel structural insights of a naturally occurring peptide isolated from the immune system of tunicates and have shown that this peptide exhibits a novel biological activity as an inhibitor of bacterial biofilms, which may have implications in enabling the sea tunicate *S. clava* to combat biofilm-related infections in its natural environment.

### Experimental procedures

#### Bacterial strains

Strains used included clinical isolates *Escherichia coli* 0157, *Klebsiella pneumonia* carabapenemase producing *K. pneumoniae* 1825971 (KPC-Kp) and methicillin resistant *Staphylococcus aureus* (MRSA) #SAP0017, as well as reference strains *E. coli* ATCC8739, *K. pneumoniae* ATCC13883 and *S. aureus* ATCC2921.

#### Peptide synthesis and mass spectrometry analyses

Clavanin A was synthesized by Peptides 2.0 (Chantilly, VA, USA) using N-9-fluorenylmethyloxycarbonyl (Fmoc) solid-phase synthesis, and purified by high-performance liquid chromatography (HPLC). The sequence and degree of purity (>95%) was confirmed by MALDI-ToF analyses^26^.

#### Bactericidal microdilution assays

Clavanin A MICs against *E. coli* ATCC8739 and *S. aureus* ATCC29213 were determined using a standardized microdilution method in polypropylene microtitre plates (TPP, Switzerland) according to CLSI guidelines, with an inoculum of 1 × 10^5^ cells per mL^34^. MICs were determined as the lowest concentration tested that led to complete inhibition (100%) after incubation at 37 °C for 24 h, in comparison with the untreated negative control group^8^,^35^.

#### Flow cell assays

Biofilms were grown in BM2 minimal medium (62 mM potassium phosphate buffer, pH 7.0, 7 mM [(NH_4_)_2_SO_4_, 2 mM MgSO_4_, 10 μM FeSO_4_] containing 0.4% (wt/vol) glucose as a carbon source, for 72 h, at 37 °C in flow cell chambers with channel dimensions of 1 × 4 × 40 mm, as previously described^36^. For the treatment of pre-formed biofilms, bacteria were allowed to develop structured 2-day-old biofilms prior to treatment with clavanin A for the following 24 h. Biofilm cells were then stained using the Live/Dead BacLight bacterial viability kit (Molecular Probes, Eugene, OR) and subsequently examined using a confocal laser scanning microscope (Olympus, Fluoview FV1000); three-dimensional reconstructions were generated using the Imaris software package (Bitplane AG).

#### Preparation of cells for transmission electron microscopy (TEM)

*E. coli* ATCC8739 and *S. aureus* ATCC29213 (1 × 10^5^) for TEM were grown and incubated with or without clavanin A (32 μg.mL^−1^ for *E. coli* and 64 μg.mL^−1^ for *S. aureus*) as described above. Cell pellets were obtained from 10 mL of each control untreated or treated cell suspension and fixed for 1 h with 2.5% glutaraldehyde in 0.1 M cacodylate buffer, pH 7.2. The samples were rinsed 2 × 10 minutes in 0.1 M cacodylate buffer, pH 7.2 and then stained for 30 min with 1% osmium tetroxide/0.8% potassium ferricyanide/5 mM CaCl_2_ in 0.1 M cacodylate buffer, and rinsed 2 × 10 min in 0.1 M cacodylate buffer. The samples were dehydrated in increasing concentrations of acetone 50%, 70%, 90% and 100% for 10 min each. After the addition of 70% acetone, the pellets were carefully removed from the bottom of the tubes with the aid of a wooden toothpick, placed in a glass Petri dish containing 70% acetone, minced into smaller pieces and transferred to glass vials containing 90% acetone and 100% acetone. The samples were infiltrated overnight with an Epon plus acetone solution (1:2); followed by 4 hr in pure Epon and embedding in fresh Epon to enable polymerization for 48 h at 60 °C. Ultra-thin sections (~70 nm) were obtained and stained for 30 min with 2% aqueous uranyl acetate and for 5 min with lead citrate. The samples were observed and the images were acquired in a JEOL JEM1011 transmission electron microscope operating at 80 KV^37^.

#### Preparation of lipid vesicles

Large unilamellar vesicles (LUV) were obtained by the extrusion method^37^. Phospholipids 1-palmitoyl-2-oleoyl-sn-glycero-3-phosphocholine (POPC) and 1-palmitoyl-2-oleoyl-sn-glycero-3-phospho-rac-(1-glycerol) (POPG) were separately dissolved in chloroform under vigorous agitation at 23 °C. Subsequently, the solutions of both pure and mixed lipid (POPC:POPG 8:2 molar) were prepared from organic stock solutions (1 mg mL^−1^) of each lipid. Next, to remove solvents the organic solution was evaporated under reduced pressure (25 min at 40 ± 1 °C) and agitation at 120 rpm. The remaining film was left under reduced pressure overnight. The dried lipid film was then hydrated by the addition of sodium HEPES buffer (pH 7.4) containing 150 mMNaCl, leading to multilamellar vesicles (MLV) formation. After that, the MLV suspension was submitted to extrusion using Hamilton 1001RN syringes (Hamilton, USA) fitted with polycarbonate membrane filters 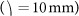 with a pore diameter of 100 nm (Nucleopore, United Kingdom) to obtain the LUV.

#### Permeability assays

Permeability was studied by detecting the clavanin A-induced leakage of 100 mM 5(6)-carboxyfluorescein (5(6)-CF) from the lipid vesicles (100 μM). The non-encapsulated 5(6)-CF was removed using size-exclusion chromatography (Econo-Pac 10 DG, Bio-Rad, USA). Then fluorescence spectra were obtained using a Varian Cary Eclipse fluorimeter (Mulgrave, Australia) in samples with 0.10 mM lipid. The measurements were recorded for 25 min at an excitation wavelength of 492 nm and emission wavelength of 517 nm^38^. The maximal release of 5(6)-CF was determined after permeabilization with 1%Triton X-100. The extent of leakage of 5(6)-CF was calculated by the equation: Leakage (%) = (F-F5)/(F_100%_-F_f_), where *F* was the fluorescence intensity measured 15 min after peptide addition, and F_5_ and F_100%_ were the fluorescence intensities measured after 5 min (to allow time for the system to stabilize) in the absence of peptide or after Triton X-100 addition, respectively. The fluorescence intensities were corrected for the dilution introduced by the addition of peptide and Triton X-100.

#### Evaluation of peptide membrane interaction by dynamic light scattering and zeta potential

The dimensions and zeta (ζ) potential of lipid (0.2 mM) vesicles were calculated using a ZetasizerNano ZS90 (Malvern, United Kingdom) with a laser He-Ne operating at a wavelength of 632.8 nm. The mean hydrodynamic diameter was determined by dynamic light scattering detected at an angle of 173°. The CONTIN analysis of the scattering correlation function was used to calculate the diffusion coefficient, and the Stokes-Einstein equation was used to convert the diffusion coefficient into the hydrodynamic diameter. Zeta (ζ)-potential (basically surface charge) was measured by an electrophoretic technique using the Smoluchowski approximation, *U* = *ε*ζ/*η*, where *U* was the mobility, *η* was the viscosity and *ε* was the dielectric constant for pure water. All reported results corresponded to the average of three independent measurements of 10 runs each carried out at 25 °C. All samples were filtered through Millipore filters with a pore diameter of 0.45 μm^39^.

#### Isothermal titration calorimetry assays

To measure the binding affinity of clavanin A towards phospholipids 1-palmitoyl-2-oleoyl-sn-glycero-3-phosphocholine (POPC) and liposome of POPC mixture with 1-palmitoyl-2-oleoyl-sn-glycero-3-phospho-rac-(1-glycerol) (POPG) isothermal titration calorimetry (ITC) was performed using the iTC200 System (GE Healthcare, USA) employing non-reactive Hastelloy cells^40^. The lipid vesicle (POPC:POPG 8:2 molar) was prepared as described above in this section. The dried lipid vesicle was hydrated with sodium HEPES buffer (pH 7.4) containing 50 mM NaCl and degassed prior to titration at 25 °C. Isothermal interactions between clavanin A and only lipid and their vesicle were measured by titrating more than 20 injections using 40 μL of peptide solution (1 mM) and lipid molecules in a sample cell at a concentration of 100 μM in a 200 μL suspension. Experiments were repeated three times.

#### Circular dichroism analyses

Circular dichroism (CD) spectra were obtained on a Jasco J-815 instrument (Jasco Co, Tokyo, Japan) using 1 mm path quartz cells. Spectra were collected from 190 to 260 nm at a speed of 50 nm.min^−1^. CD spectra were obtained with 41 mM peptide in the presence of different concentrations of SDS (sodium dodecyl sulphate) and TFE (2,2,2-trifluorethanol). The effect of variation of pH was analyzed using 5 mM sodium acetate buffer at pH 3, 4 and 5, and 5 mMTris-HCl buffer at pHs 7 and 9. Spectra were corrected by subtracting the appropriate blank control and converting to mean residue molar ellipticity. Analyses of percentage of 〈-helicity were performed according to the method by Chen *et al*.^41^.

#### Nuclear Magnetic Resonance

The NMR sample was prepared by dissolving clavanin A to a final concentration of 2 mM in 500 μL of 35% (v/v) TFE-*d*_*3*_ solution, 10% of D_2_O and pH 4.3. Spectra were acquired at 25 °C on a Bruker Avance III 500 spectrometer operating at 11.75 T. All acquired NMR spectra were referenced using TMSP-2,2,3,3-d_4_ (sodium-3-trimethylsilylpropionate). ^1^H–^1^H Total Correlation Spectroscopy (TOCSY) spectra were recorded with 176 transients of 4096 data points, 256 t_1_ increments and a spinlock mixing time of 80 ms, using the dipsi2esgpph^42^ pulse sequence. The ^1^H–^1^H Nuclear Overhauser Effect Spectroscopy (NOESY) was recorded with 64 transients of 4096 data points, 256 t_1_ increments, mixing time of 250 ms, using the noesygpphw5^43^ pulse sequence. ^1^H–^15^N So-fast Heteronuclear Multiple Quantum Coherence (sf-HMQC; sfhmqcf3gpph pulse sequence)^44^ and ^1^H–^13^C Heteronuclear Single Quantum Coherence spectra (HSQC; hsqcetgp pulse sequence) were recorded with 168 transients of 1024 data points and 256 t_1_ increments. ^1^H–^1^H TOCSY, NOESY and ^1^H–^15^N sf-HMQC spectra were acquired using the States-TPPI mode^45^ and ^1^H–^13^C HSQC spectra were acquired with the Echo-antiecho^46^ mode of quadrature detection. All NMR data were processed using nmrPIPE^47^ and nmrVIEW^48^ software. The complete assignment of the backbone and side-chain ^1^H resonances, to accessible protons, of clavanin A was performed using standard sequential assignment procedures, according to the methodology developed by Wüthrich^49^. The chemical shifts were deposited in the BioMagResBank ( www.bmrb.wisc.edu) under accession number 25262. Clavanin structure was also deposited in Protein Databank (Code: 2MVE) and validation was described in Supplementary Material 1.

#### Molecular Modelling

The volumes of NOE correlations from NOESY were converted into distance using XPLOR-NIH (version 2.28) software. The restraints were classified into short (2.8 Å), medium (3.4 Å) and long (5.0 Å) distances and analyzed using QUEEN (Quantitative Evaluation of Experimental NMR Restraints) software^50^. Dihedral angles were predicted using the software TALOS+, where the chemical shift constraints of ^13^C, ^15^Ne ^1^H were used as input^12^. Structures were calculated using the XPLOR-NIH and the simulated annealing (SA) algorithm^51^. The structure calculation started with an extended model, with 18,000 steps at high temperature, and 9,000 steps of cooling. The ensemble of 20 lower energy structures (from a total of 200 calculated structures) was then submitted to a water refinement protocol. The ensemble of 10 lowest energy structures was chosen to represent the peptide solution 3D structure. The generated structures were validated using Procheck online software (mordred.bioc.cam.ac.uk/~rapper/rampage.php). The quality of the family of structures was analyzed by standard measures used to quantify differences between three-dimensional structures, namely the root-mean-square deviation (RMSD), provided by the MOLMOL program^52^. The NMR structures were submitted to PDB and were assigned with PDB code 2MVE.

#### Molecular docking and dynamics

The complexes between clavanin A and lipids DOPC or DPPC were constructed using the Hex 6.1^53^. The NMR structure was used for docking; and the coordinates of DOPC and DPPC were obtained from the model CHARMM-GUI server^54^. Docking experiments were performed with consideration to the shape and electrostatics of each molecule without any docking post processing. The resulting complexes were clustered, using a root mean square (RMS) cut-off of 3 Å. The cluster with highest affinity was selected as the preferable binding mode. For molecular dynamics (MD), the structures of DOPC and DPPC were parameterized using the PRODRG server^55^. The GROMACS package (version 4.5)^56^ was used for performing the MD simulations. The structures in complex with, or without, lipid were immersed in a cubic water box, with a distance of 0.7 nm from the edges of the box. Water molecules were represented using the single point charge water model^57^. Chlorine ions were added to the system in order to neutralize the system’s charge. Fifty thousand steps of steepest descent were performed to minimize the system. Next, the molecular dynamics integrator was used for pressure and temperature normalization (100 ps each) by using the velocity rescaling thermostat (NVT ensemble) and the Parrinello-Rahman barostat (NPT ensemble), respectively. Then, the system was simulated for 50 ns with minimized energy and normalized pressure (1 bar) and temperature (300 K). The geometry of water molecules was constrained by using the SETTLE algorithm^58^ and all atom bond lengths were linked by using the LINCS algorithm^59^. The electrostatic corrections were made according to the Particle Mesh Ewald algorithm^60^, with a cut-off radius of 1.4 nm; the same cut-off radius was also used for van der Waals interactions. The MD simulations were analyzed by means of root mean square deviation (RMSD) and standardize secondary structure assignment (DSSP).

## Additional Information

**How to cite this article**: Silva, O. N. *et al*. Structural Studies of a Lipid-Binding Peptide from Tunicate Hemocytes with Anti-Biofilm Activity. *Sci. Rep.* **6**, 27128; doi: 10.1038/srep27128 (2016).

## Figures and Tables

**Table 1:** ζ-potential values for POPC and POPC:POPG at 100 μM vesicles before and after clavanin A exposition.

Sample	Peptide	⌠-potential (mV)
POPC	None	−1.53 ± 0.20
POPC	Clavanin	−0.65 ± 0.43
POPC:POPG	None	−19.80 ± 0.56
POPC:POPG	Clavanin	−19.40 ± 1.76

**Table 2:** Permeabilization of liposomes and exposure to different concentrations of clavanin A.

Clavanin concentration (μg.ml^−1^)	Permeabilization of Liposomes (%)	
	POPC	POPC:POPG
3	98.3	92.3
13	98.8	98.7
27	99.4	100.0

**Table 3:** ITC binding experiments between clavanin A and DOPC, DPPC and ergosterol.

Parameter	Thermaltransition		
	DOPC (**a**)	DPPC (**b**)	Ergosterol (**c**)
**K**	1.31 × 10^5 ^M^−1^	1.56 × 10^3 ^M^−1^	No interaction
Δ**H**	6.86 E^6 ^cal/mol	5.93 E^5 ^cal/mol	–
Δ**S**	2.08 E^4 ^cal/mol/deg	1.76 E^3 ^cal/mol/deg	–

Thermal transition data derived from raw heat associated isotherm using nonlinear regression.

**Table 4:** Statistics for the 10 lowest energy structures of clavanin A.

NOE RESTRAINTS	
Total number of distance restraints	283
Number of intra-residue restraints	138
Number of sequential restraints (*i*, *i* + 1)	81
Number of medium range restraints (*i*, *i* + j)_j=2,3,4_	22
Number of long range restraints (|i–j| >5)	0
HydrogenBonds	0
Dihedralangles	42
RMSD^a^	Ǻ
All heavy atoms	1.99 ± 0.41
Allbackboneatoms	1.05 ± 0.34

^a^DatafromPyMol.
